# Canine babesiosis and tick activity monitored using companion animal electronic health records in the UK

**DOI:** 10.1136/vr.103908

**Published:** 2016-08-02

**Authors:** F. Sánchez-Vizcaíno, M. Wardeh, B. Heayns, D. A. Singleton, J. S. P. Tulloch, L. McGinley, J. Newman, P. J. Noble, M. J. Day, P. H. Jones, A. D. Radford

**Affiliations:** 1 Institutes of Infection and Global Health University of Liverpool, Leahurst Campus, Chester High Road, Neston, S. Wirral CH64 7TE, UK; 2 Veterinary Science, University of Liverpool, Leahurst Campus, Chester High Road, Neston, S. Wirral CH64 7TE, UK; 3 University of Bristol, School of Veterinary Sciences, Langford, Bristol BS40 5DU, UK

**Keywords:** Babesiosis, Dogs, Tickborne diseases, Ticks

## Abstract

Recent publications highlighting autochthonous *Babesia canis* infection in dogs from Essex that have not travelled outside the UK are a powerful reminder of the potential for pathogen emergence in new populations. Here the authors use electronic health data collected from two diagnostic laboratories and a network of 392 veterinary premises to describe canine *Babesia* cases and levels of *Babesia* concern from January 2015 to March 2016, and the activity of ticks during December 2015–March 2016. In most areas of the UK, *Babesia* diagnosis in this population was rare and sporadic. In addition, there was a clear focus of *Babesia* cases in the affected area in Essex. Until February 2016, analysis of health records indicated only sporadic interest in *Babesia* largely in animals coming from overseas. Following media coverage in March 2016, there was a spike in owner concern that was geographically dispersed beyond the at-risk area. Tick activity (identified as ticks being removed from animals in veterinary consultations) was consistent but low during the period preceding the infections (<5 ticks/10,000 consultations), but increased in March. This highlights the use of electronic health data to describe rapidly evolving risk and concern that follows the emergence of a pathogen.

## Introduction

Canine babesiosis is caused by several species of an intraerythrocytic protozoan parasite that causes haemolytic disease (Irwin 2009, Cook and Swann 2016). Each species is tick transmitted, and generally maintained by transovarial and trans-stadial transmission. The geographical distribution of canine babesiosis is therefore largely driven by the habitat of relevant tick vector species, and in Europe has generally been limited to the mainland (Solano-Gallego and Baneth 2011). In the UK, competent vector species were largely considered to be absent, such that until recently, cases were sporadic and generally restricted to animals returning to the UK under the PETS travel scheme (Shaw and others 2003). However, a single fatal case of *Babesia vogeli* infection reported in 2006 in a dog living in Ashford, (county of Kent, England) that had no travel history, highlighted the potential for populations of ticks in the UK to establish endogenous infection (Holm and others 2006). *B vogeli* is transmitted by *Rhipicephalus sanguineus* ticks, which were known at that time to be present around UK quarantine kennels, in houses and in vehicles (Hoyle and others 2001). More recently, in February 2016, Swainsbury and others (2016) reported three further cases of babesiosis seen in one Essex Veterinary practice during the preceding three months in dogs that also had not travelled abroad. All the affected dogs were exercised in a common area of uncultivated park within Harlow. Subsequently, a tick from one of the affected dogs was identified as *Dermacentor reticulatus*, and was shown to be carrying *Babesia canis* (Phipps and others 2016). These publications were picked up by the media locally on the March 13, 2016 (BBC 2016a), leading to national coverage on the March 16, 2016 (BBC 2016b, Guardian Online 2016).

Such cases are a powerful reminder of how patterns of disease in populations can evolve rapidly, and highlight the need for surveillance systems to monitor them, and facilitate their prevention and control (Matjila and others 2005). Here, the authors use electronic health data from both veterinary surgeons and diagnostic laboratories to provide a novel national perspective on the status of *Babesia* in the UK focusing on *Babesia* diagnosis in dogs, tick activity (as measured by the presence of ticks on animals recorded during consultations) and *Babesia* concern.

## Materials and methods

Canine *Babesia* diagnosis: the results of *Babesia* diagnostic testing at the genus level from two diagnostic laboratories Idexx (Wetherby, UK) and Torrance Diamond Diagnostic Services (Exeter, UK) that carry out PCR assays were collated through the Small Animal Veterinary Surveillance Network (SAVSNET). Data are received daily (near real-time). For each sample tested, the species, date of sample receipt at the laboratory, test result at the genus level and the postcode area (first one or two letters of the postcode) of the submitting practitioner are also captured. All tests were recorded between January 2015 and March 2016. The samples which tested positive for *Babesia* were mapped using QGIS V.2.8.2-Wien.

Tick activity and *Babesia* concern: electronic health records were collected in real-time from 392 volunteer veterinary premises (sites) across the UK. These sites are chosen based on convenience, largely because of their use of compliant practice management software systems; currently RoboVet and Teleos. Electronic health data were gathered at the end of individual consultations in real time and included the postcode of the owner, the date of the consultation, as well as the free-text field (clinical narrative) written by the attending veterinary practitioner or nurse. Clinical narratives from December 1, 2015 to March 31, 2016 containing the word ‘tick’, but not ‘tickl’, ‘ticki’ or ‘stick’, were identified using a simple free-text analysis approach. Positive consultations were then read by a domain expert using a strict case definition to identify only those consultations where ‘a veterinary surgeon or nurse confirmed visual sighting or removal of a tick within the consultation’. Reference to the historical presence of ticks was excluded from the analyses. Potential ticks that were only seen by owners were also excluded as it was clear from reading the clinical narrative that many owners misidentify skin lesions for ticks (data not presented).

A similar simple text mining approach was used to identify those consultations from January 2015 and March 2016 where reference to *Babesia* was recorded using the search term ‘*Babesia*’. Consultations were also coded as to whether the concern over *Babesia* related to an overseas or UK risk of infection.

The 95 per cent confidence interval for the proportion of all consultations in which a tick was referred to was calculated using robust standard errors to allow for the clustering within veterinary premises. These estimates were carried out using R language (V.3.2.0). The spatial distribution of the tick activity and Babesia concern was depicted using QGIS V.2.8.2-Wien.

Ethical approval for this study was received from the University of Liverpool Ethics Committee (000964).

## Results

**FIG 1:**
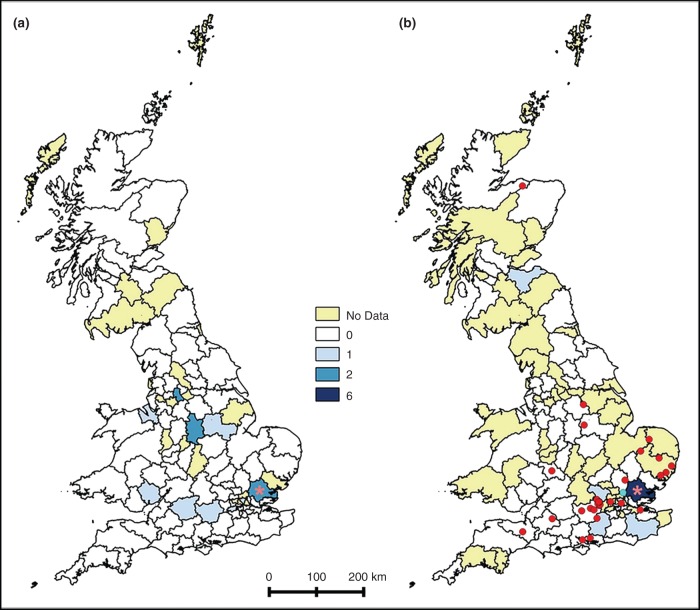
Samples testing PCR positive for *Babesia* in (a) 2015 and (b) January–March 2016. The red dots in (b) indicate the locations of owners expressing concern about UK risk of *Babesia* infection in February–March 2016. The red asterisk indicates the Chelmsford postcode area, the location of the initial outbreak (Swainsbury and others 2016). The yellow areas indicate postcode areas from which no diagnostic samples were received from participating laboratories during the study period

Samples were submitted for diagnosis of canine babesiosis by PCR to the two laboratories used in this study from a total of 101 of the 121 UK postcode areas including 99 in 2015 and 67 in January–March 2016 (Fig 1). A total of 24 samples tested positive, including 13 from 2015 (Fig 1a), and 11 in the first three months of 2016 (Fig 1b). The highest number of positive samples originated from veterinary practices in the Chelmsford (CM) postcode area, where the published cases were identified, including one each in May and October 2015, and three, one and two cases in January, February and March 2016, respectively (total eight positives). For postcode areas submitting more than 10 samples during the study period, the CM postcode area also had the highest proportion testing positive (8 of 21 submissions; 38.1 per cent).

**FIG 2:**
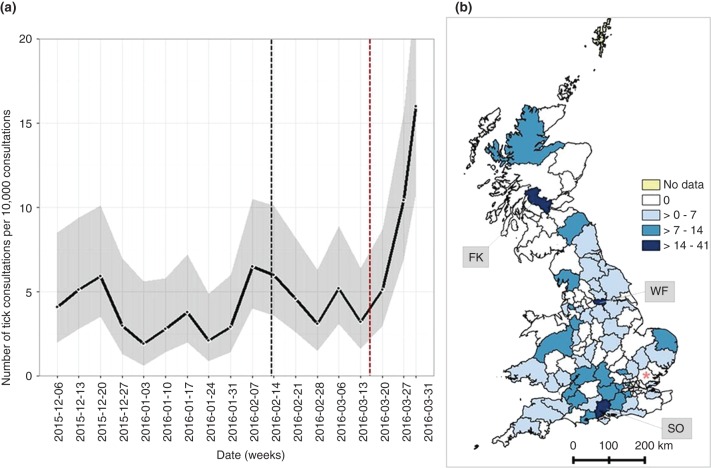
Tick activity in the UK between December 2015 and March 2016 based on reference to ticks in the clinical narrative. (a) Seasonality of ticks by week. The grey shadow shows 95 per cent confidence intervals calculated in each week. The dotted lines in black and red indicate the week of the first publication describing three autochthonous cases (Swainsbury and others 2016), and the week of the national media coverage respectively. (b) Number of tick consultations per 10,000 consultations in each postcode area. SO (Southampton), FK (Falkirk), WF (Wakefield). The red asterisk indicates the Chelmsford postcode area, the location of the initial outbreak

In total 1779 of 395,210 (0.4 per cent) consultations collected between December 1, 2015 to March 31, 2016 had a clinical narrative containing the word ‘tick’. Of these, 196 (11.0 per cent) consultations were identified as having a confirmed tick present in the consultation when read by a domain expert. All weeks were associated with a low level of tick activity (Fig 2a). For all but the last two weeks of the study period, ticks were identified in fewer than 8/10,000 consultations; there was some evidence to suggest a significant increase in tick detection at the end of the study period. The spatial distribution of these 196 consultations as a proportion of the overall number of consultations in each postcode area is shown in Fig 2b. Ticks were clearly active over large areas of the UK; the areas with the highest tick activity were Southampton, Wakefield and Falkirk. None of the 1973 consultations collected from 17 premises in the CM postcode area between December 2015 and March 2016 were associated with tick removal.

In total between January 2015 and March 2016, 59 of 837,141 consultations (0.7/10,000 consultations) had a clinical narrative containing the word *Babesia*, including 9 from 2015, 2 from January 2016, 1 from February 2016 and 47 in March 2016. None of these concerns related to confirmed cases of *Babesia* (note the practice reporting the autochthonous cases is not currently part of SAVSNET). Of the 11 consultations occurring up to and including January 2016 before the *Babesia* media coverage, 8 were clearly associated with overseas travel, none expressed concern about UK risk of infection, with the rest not specifying a geographical concern. Of the 48 consultations occurring in February–March 2016, 3 expressed concern about overseas risk, and 33 with UK risk, 32 of which followed the national media coverage on March 16, 2016. The location of these 33 owners expressing concern about UK risk is shown in Fig 1b. Of the 10 consultations identified over the entire study period where overseas risk was clearly mentioned, two referred to France and one each to Turkey, Spain, Greece, Thailand and Cyprus, with three others just referring to overseas travel.

## Discussion

The emergence of a new disease in a geographical location is often followed by a period of uncertainty driven by incomplete data and evidence. Such an emergence of canine babesiosis was rapidly brought to the attention of the veterinary profession by the timely and thoughtful clinical work of Swainsbury and others in one veterinary practice that linked together their first three cases of canine babesiosis in non-travelled dogs (Swainsbury and others 2016). Here the authors have used a large-scale collection of electronic health data collected in real-time and near real-time from a convenience-based sample of veterinary practices and diagnostic laboratories respectively, to provide additional insight into *Babesia* infection in the UK, and more specifically the recent emergence of autochthonous *B canis* in dogs in the south-east of England.

Taking data from the clinical narrative on *Babesia* concern, together with results of *Babesia* diagnosis from diagnostic laboratories, the authors have defined a sporadic and low background level of *Babesia* infection in the UK that is likely to be largely associated with overseas risk. Based on text mining of the clinical narrative, it was clear that before this outbreak was reported first in February 2016, *Babesia* was only rarely and sporadically recorded as being discussed in the veterinary consultation, and when it was, it usually related to overseas travel. Over the same time period, canine samples were also being regularly submitted from large areas of the UK to two diagnostic laboratories for *Babesia* diagnosis by PCR. In total, eight postcode areas submitted 13 positive samples in the year 2015. Since SAVSNET does not collect data from all diagnostic laboratories, the total UK *Babesia* cases confirmed in dogs may be higher. Whilst the authors are not able to confirm the travel history of these cases, it seems reasonable based on their sporadic and geographically distributed nature, together with the lack of published evidence of previous foci of autochthonous infection, that these cases also mostly represent animals coming to the UK from overseas. However, clearly rare cases of UK acquisition cannot be ruled out. These sporadic cases of canine babesiosis represent a background against which any potential outbreak needs to be considered.

Laboratory data also confirmed a cluster of *Babesia* cases in the CM area of Essex, the same postcode area as the four autochthonous cases of *Babesia* reported in the literature (Phipps and others 2016, Swainsbury and others 2016). Here, the authors identified a total of eight cases from this same postcode area, the earliest in May 2015. Some of these cases are likely to be the same as those published, but the collection of partial postcode data did not allow the authors to confirm this. Unfortunately, it is not possible with these kinds of laboratory data to know the travel history of the animals being tested. However, the clustering of these cases does suggest an increased risk for the local dog population, as would be associated with exposure to a local infected tick population. The authors cannot however rule out that the growing cluster of cases in this postcode area may also reflect: (i) increased diagnostic sensitivity by local veterinary surgeons who are likely to be most aware of the *Babesia* risk to dogs in this area, or (ii) to high levels of overseas travel by dogs from this area to known *Babesia*-endemic areas.

An important question to answer is why these autochthonous cases have come to light now. The clinical signs of babesiosis are often quite severe (Cook and Swann 2016). It therefore seems likely that most cases in owned dogs would seek veterinary care. However, the growing reliance on automated haematological analysers, together with the low index of submission for *Babesia* in non-travelled dogs, could mean some autochthonous cases are being missed nationally. In the current outbreak, it seems more likely that either the infected ticks have recently arrived, such as an infected female tick dropping off a dog, laying eggs which produce infected larvae, or the ticks have either recently become infected, or recently gained regular contact with dogs in the area. Previous publications make it clear that the vector *D reticulatus* exists in pockets in the UK, and has previously been identified in Essex in Southend, only about 60 km from the source of the current outbreak (Jameson and Medlock 2009). It therefore seems probable that these tick populations have existed in local foci for some time. Any sporadic imports of *B canis* infected dogs could have recently established a local infection when the tick vector fed on parasitised dogs.

The incubation period of *Babesia* in dogs is suggested to be 10–28 days (Boozer and Macintire 2003). This would place the infection date for the six cases the authors identified in 2016 in the CM area between December 2015 and March 2016. Although the authors’ data do not allow them to speciate ticks identified in consultations, the results of the present study do make it clear that ticks were active in the UK throughout this winter period. It is striking that ticks remain active in winter, albeit at presumably low levels, and also that this outbreak was reported at a time of seemingly lowest risk. The coming rising temperatures and tick activity will pose an increased risk for autochthonous infection in this location. Interestingly, ticks were not recorded in consultations from the CM postcode area of the outbreak, but that may be associated with the relatively few consultations currently received each month from that area.

In almost all cases, veterinarians removing ticks in consultations did not indicate the species of the tick in the clinical narrative. Since *D reticulatus* can have a distinct morphology, particularly when unfed, this is something veterinarians could perhaps be encouraged to report in their electronic health records if seeing ticks that are not engorged. This could allow tick surveillance by real-time electronic health records to better identify populations of dogs at risk from individual tickborne diseases. It would also complement existing tick surveillance activities such as Public Health England's Tick Recording Scheme (Public Health England 2016) or the Big Tick Project (University of Bristol).

Following the first reports of *Babesia* emergence in veterinary literature in the UK (Swainsbury and others 2016), there was not surprisingly a proliferation of concern as evidenced by increased *Babesia* discussion recorded in consultations, and it was clear that this closely followed the media coverage focusing on the risk of acquiring *Babesia* in the UK. This raised awareness is a vital part of the response to an outbreak. However, this increased awareness seemed poorly focused on the precise risk area, possibly reflecting the national press coverage. Whilst the veterinary literature did report the geographical focus of the outbreak, the national media did not highlight it, using headlines like ‘Dog owners in the UK are being warned about an outbreak of an animal disease that is carried by ticks’ (BBC 2016b) and often highlighting the fact the disease will likely spread ‘Tick-borne disease that can kill dogs will spread in UK, experts warn’ (Guardian Online 2016). Focusing behavioural change to the high-risk populations is a key component of effective outbreak control.

In conclusion, following the initial dedicated work of the veterinary practice team in CM, the authors have confirmed the emergence of an increased *Babesia* risk in dogs attending veterinary practices around the CM area of the UK; this increased risk is likely to be significantly contributed to by autochthonous infection. In addition to this, a low level of sporadic cases were also diagnosed throughout the UK, which the authors hypothesise are more likely to be associated with overseas risk. Ticks remain active throughout the winter in the UK. Practitioners should be aware of this localised risk of autochthonous *Babesia* infection, and are in the best place to focus the early enthusiasm of the media, to ensure the most appropriate local response. Practitioners should remind themselves of such tick-transmitted diseases. Health informatics surveillance conducted by SAVSNET can now provide real-time local updates on this and other important pathogens (www.savsnet.co.uk/realtimedata), monitor the response to such outbreaks and in the future contribute to their early detection.
